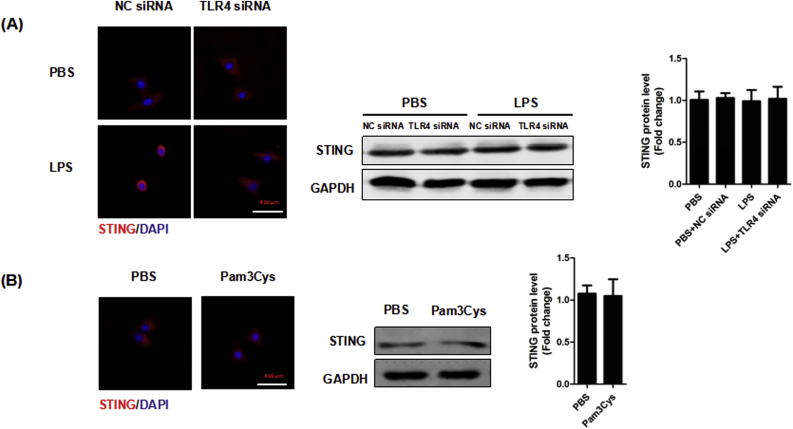# STING-IRF3 contributes to lipopolysaccharide-induced cardiac dysfunction, inflammation, apoptosis and pyroptosis by activating NLRP3

**DOI:** 10.1016/j.redox.2019.101215

**Published:** 2019-05-13

**Authors:** Ning Li, Heng Zhou, Haiming Wu, Qingqing Wu, Mingxia Duan, Wei Deng, Qizhu Tang

**Affiliations:** Department of Cardiology, Renmin Hospital of Wuhan University, Hubei Key Laboratory of Metabolic and Chronic Diseases, Wuhan, 430060, PR China

**Keywords:** STING-IRF3, NLRP3 inflammasome, Pyroptosis, Apoptosis, Sepsis-induced cardiomyopathy

## Abstract

Mountainous evidence suggests that inflammation, cardiomyocyte apoptosis and pyroptosis are involved in the development of sepsis and sepsis-induced cardiomyopathy (SIC). Stimulator of interferon genes (STING) is an indispensable molecule that could regulate inflammation and immune response in multiple diseases. However, the role of STING in cardiovascular disease, especially SIC remains unclear. This study was designed to investigate the potential molecular mechanisms of STING in lipopolysaccharide (LPS)-induced cardiac injury using STING global knockout mice. In wild type mice and cardiomyocytes, LPS stimulation triggered the perinuclear translocation of STING, which further bound to Type-I interferons (IFN) regulatory factor 3 (IRF3) and phosphorylated IRF3. Phosphorylated (P-) IRF3 subsequently translocated into nucleus and increased the expression of NOD-like receptor protein 3 (NLRP3). Knockout of STING in mice significantly improved survival rate and cardiac function, apart from suppressing myocardial and serum inflammatory cytokines, apoptosis, as well as cardiomyocyte pyroptosis. *In vitro* experiments revealed that NLRP3 overexpression by adenovirus could offset protective effects of STING knockdown in LPS-induced cardiomyocytes. Additionally, LPS stimulation also promoted the production of intracellular reactive oxygen (ROS), which further induced the NLRP3 translocation to the cytoplasm from the nucleus. Dissociative TXNIP could directly interact with cytoplasmic NLRP3 and form inflammasome, eventually triggering cardiomyocyte injury. Collectively, our findings disclose that STING deficiency could alleviate LPS-induced SIC in mice. Hence, targeting STING in cardiomyocytes may be a promising therapeutic strategy for preventing SIC.

## Highlights

• STING-IRF3 could regulate inflammation and immune response in multiple diseases.
• NLRP3 inflammasome is involved in the development in sepsis-induced cardiomyopathy.
• STING-IRF3 triggers cardiac injury by activating NLRP3 inflammasome in sepsis-induced cardiomyopathy.

## 1 Introduction

Sepsis is a lethal syndrome caused by a series of inappropriate immune responses, which has been estimated to cause annual death of 200,000 in America [1]. Sepsis may develop into systemic inflammatory response syndrome (SIRS) unless it is controlled properly and promptly, eventually giving rise to multiple organ dysfunction (MOD) [2]. Sepsis-induced cardiomyopathy (SIC) is one of pervasive and well-described complications during sepsis and septic shock. The earliest clinical manifestation in patients with SIC is cardiac dysfunction, which has 3 features: dilated left ventricle, declined ejection fraction, as well as recovery in 7–10 days [3]. At the cellular and molecular level, inflammation, apoptosis and pyroptosis are regarded as critical pathophysiology phenomenons in sepsis and SIC [[4], [5], [6]]. Therefore, molecules or genes which could selectively suppress the above processes would be of great therapeutic interest in sepsis and SIC.

The NOD-like receptor protein 3 (NLRP3) inflammasome, a vital mediator pathway of the innate immune system, has gained close attention in SIC recently. NLRP3 inflammasome components mainly include 3 members, namely apoptosis-associated speck-like protein (ASC), NLRP3 and procaspase-1 [7]. Thioredoxin-interacting protein (TXNIP), an endogenous inhibitor of reactive oxygen (ROS) scavenging protein thioredoxin, will be dissociated from TXNIP-thioredoxin (Trx) protein complex and bind NLRP3 when cells are challenged with ROS induced by pathogen-associated molecular patterns (PAMPs) and damage-associated molecular patterns (DAMPs) [8]. Subsequently, NLRP3/TXNIP complex recruits other 2 members (ASC and procaspase 1) and forms NLRP3 inflammasome, contributing to activation of caspase-1, production of IL-18 and IL-1β, and initiation of inflammation and pyroptosis [9]. Recent studies proved that NLRP3 inflammasome triggered the cardiomyopathy of polymicrobial sepsis induced by cecal ligation and puncture [10]. But the precise regulatory mechanisms of NLRP3 in SIC remain incompletely clear.

Stimulator of interferon gene (STING), which is comprised of 5 putative transmembrane regions, mainly resides in the endoplasmic reticulum and could activate both nuclear factor (NF)-κB and interferons (IFN) regulatory factor 3 (IRF3) transcription pathways to up-regulate the level of type I interferon [11]. STING displays critical pro-inflammatory and immunoregulatory effects in multiple diseases ranging from cardiovascular disease to liver disease. In alcoholic liver disease, increased gut permeability resulted by ethanol gives rise to the accumulation of lipopolysaccharide (LPS) in liver tissue and enhanced endoplasmic reticulum stress in hepatocytes. The stress will further trigger the dissociation between STING and endoplasmic reticulum, which subsequently activates IRF3 and induces hepatocyte apoptosis [12]. In human myeloid cell infected by viral and bacterial, NLRP3 is hardwired to the upstream DNA-sensing cGAS-STING pathway to induce inflammasome activation and orchestrate a lysosomal cell death program in a K^+^ efflux dependent manner [13]. However, the roles of STING as well as its potential regulatory effects on NLRP3 in SIC have not been well investigated till now.

Therefore, the aims of this study were to determine whether STING could affect LPS-induced cardiac dysfunction and injury, and meanwhile to elucidate the potential mechanisms of STING in SIC.

## 2 Methods

### 2.1 Materials

Lipopolysaccharide was obtained from Sigma (St Louis, MO, USA). IRF3 agonist, KIN1148 and ROS scavenger, N-acetyl-l-cysteine (Nac) were purchased from Medchem Express (Shanghai, China). IRF3 siRNA, STING siRNA and the scrambled siRNA were acquired from Santa Cruz Biotechnology (Dallas, TX, USA). Primary antibodies against NLRP3, STING, phospho-IRF3 (P-IRF3), total-IRF3 (T-IRF3), CD45, CD68, IL-18, BCL-2, BAX, TXNIP, ASC and Trx were purchased from Abcam (Cambridge, UK). Primary antibodies for Caspase1 and IL-1β were purchased from Proteintech Group (Rosemont, USA). Primary antibodies against proliferating cell nuclear antigen (PCNA), GAPDH, C-Casepase3 and Caspase3 were acquired from Santa Cruz Biotechnology (Dallas, TX, USA). Pam3Cys-Ser-(Lys)4 was purchased from Abcam (Cambridge, UK). The GTVision™^ ^+ Detection System/Mo&Rb reagent was obtained from Gene Technology (Shanghai, China). The Alexa Fluor 488- and 568-goat anti-rabbit secondary antibodies used in immunofluorescence staining were purchased from LI-COR Biosciences (Lincoln, USA). Lactate Dehydrogenase Assay Kit (Colorimetric) and Cell Counting Kit 8 (WST-8/CCK8) were obtained from Abcam (Cambridge, UK). Other chemicals in this study were of analytical grade.

### 2.2 Animals

All animal experiment procedures in this study were approved by the National Institutes of Health (NIH) Guide for the Care and Use of Laboratory Animals (Revised 2011) as well as the guidelines of Renmin Hospital of Wuhan Hospital (approval number: WDRX-2017K012). The entire experimental process was carried out in a blinded manner all along, involving the animal models and subsequent analyses.

Male C57/B6 mice (8–10 weeks old) were purchased from the Institute of Laboratory Animal Science, Chinese Academy of Medical Sciences (Beijing, China). Male STING−/− mice (8–10 weeks old, C57BL/6 background) were obtained from Jackson Laboratory (California, USA). All mice were housed in a specific-pathogen-free (SPF) environment (humidity 50 ± 5%; temperature 20–22 °C). Sepsis-induced cardiomyopathy mice models were induced by LPS injection intraperitoneally (10 mg/kg) as described by our previous study [14]. The sham groups were given an intraperitoneal injection with an isovolumetric saline. 12 h later, mice were anesthetized and transthoracic echocardiography was carried out to determine their cardiac function. Then, mice were sacrificed with sodium pentobarbital (200 mg/kg). The myocardial tissues were snap-frozen in liquid nitrogen for further measurement.

### 2.3 Echocardiography

As described [15], M-mode echocardiograms were implemented by a MyLab 30CV ultrasound (Esaote SpA, Genoa, Italy), which carried a 10 MHz phased array transducer. To begin with, mice were anesthetized with 1.5% isoflurane and then subjected to echocardiographic examination. Then, the parameters of cardiac function including ejection fraction (EF), fractional shortening (FS), left ventricle (LV) end-systolic diameter (LVEDd), LV end-diastolic diameter (LVEDs), LV end-systolic posterior wall thickness (PWs) and LV end-diastolic posterior wall thickness (PWd) were collected.

### 2.4 Survival condition

The extra mice in each group (n = 10) that had free access to water and food were kept to observe their survival condition. The number of death was recorded at the same time point every day. The percent survival was calculated within 7 days after the sham or LPS injection (10 mg/kg).

### 2.5 LDH and CK MB release in serum

Blood samples in heparinized tubes from each group were centrifuged at 3,000×*g* for 15 min (4 °C). After then, the plasma samples were stored at −80 °C for the subsequent analyses. The enzyme activity of lactate dehydrogenase in the serum was measured using a quick, convenient, and sensitive LDH assay kit based on the protocol [16]. Serum concentration of creatine kinase isoenzymes (CK MB) was detected by an automatic biochemical analyzer (ADVIA^®^ 2400, Siemens Ltd., China).

### 2.6 Inflammatory cytokines in serum

Inflammatory cytokines in this study included IL-1β, TNF-α, MCP-1 and HMGB1. These cytokines in serum were detected using commercially available ELISA kits from Abcam on the basis of the manufacturer's instructions.

### 2.7 Histological analysis

Hematoxylin&eosin (H&E) staining, Tunel staining and immunohistochemical staining were performed following previously described [17]. Briefly, fixed myocardial tissues were firstly dehydrated and embedded in paraffin. The hearts transversely sectioned from middle segment were performed to H&E for the measurement of inflammatory cells infiltration and cardiomyocyte morphology. Tunel staining was performed based on the standard protocol by ApopTag^®^ Plus Fluorescein In Situ Apoptosis Detection Kit (Millipore, USA) as described previously [18]. The apoptotic rate of cardiomyocytes was defined as the ratio of the number of Tunel-positive cells to the total number of cells. Immunohistochemical staining was performed for further assessing the content of inflammatory cells and Caspase1 using GTVisionTM + Detection System/Mo&Rb (GK600710) on the basis of standard protocol. At room temperature, the nonspecific binding of the antibody and endogenous peroxidase were blocked with H_2_O_2_ (3%) for 20 min and goat serum (10%) for 1 h, respectively. Then, the sections were incubated with anti-CD68 (1:200), anti-CD45 (1:100), or anti-Caspase1 (1:100) overnight at 4 °C. Subsequently, these sections were incubated with an anti-rabbit EnVisionTM +/HRP reagent (37 °C, 1 h) and DAB (room temperature, 5 min). Finally, these sections were observed using the light microscopy [Nikon (Tokyo, Japan), H550L].

### 2.8 Cell culture and treatment

Neonatal rat cardiomyocytes (NRCMs) from rat (1–2 days old) left ventricle were isolated and cultured according as described in our previous study [19]. NRCMs were transfected with adenovirus (Ad-) to overexpress NLRP3 (MOI = 50) or STING (MOI = 50) for 6 h. H9c2 cells were obtained from the Cell Bank of the Chinese Academy of Sciences (Shanghai, China). H9c2 cells without mycoplasma pollution were used in subsequent experiments. To knock down certain target genes, cells were incubated with STING siRNA, IRF3 siRNA, TLR4 siRNA and the scrambled RNA. All cells in this study were transfected by Lipofectamine 3000 (Thermo Fisher Scientific) following the manufacturer's instructions. The control groups were exposed to normal DMEM/F12 (Gibco, C11330) with fetal bovine serum (15%, HyClone). When the cells reached 75% confluence, LPS (1 μg/ml) was added to the medium to construct a LPS-induced cardiomyocyte injury model in vitro. After LPS challenge for 6 h, the cardiomyocytes which were seeded in 6-well plates were harvested for protein detection and RNA analysis, in 24-well plates for immunofluorescence staining analysis and in 96-well plates for ELISA measurement. Samples in one experiment indicated an independent replicate, and each experiment in our study was repeated at least 3 times.

### 2.9 Western blot and real-time PCR

To begin with, the total proteins in frozen ventricle tissues and iced cell lysates were extracted and quantified by RIPA agent (Invitrogen, Carlsbad, CA, USA). According to previous protocol [20], cytosolic protein and nuclear protein fractions were separated using a commercial kit (Thermo Fisher Scientific). 50 μg of total proteins were resolved on an SDS/PAGE gel and were subsequently transferred to polvinylidene fluoride (PVDF) membranes (Millipore, Billerica, MA, USA). The membranes were blocked by 5% none-fat dry milk with TBS containing 0.1% Tween-20 for 1 h and incubated with primary antibodies with gentle agitation overnight at 4 °C. After incubation with secondary antibodies conjugated to IRDye 800CW for 1 h at room temperature, the proteins were screened and quantified using the Odyssey infrared imaging system (Odyssey, LI-COR, Lincoln, NE). Nuclear proteins were normalized to PCNA while cytosolic proteins were normalized to GAPDH.

The total RNA was isolated from the frozen ventricle tissues or NRCMs cells using TRIzol reagent (Invitrogen, 15596-026) and reverse-transcribed to cDNA using a Transcriptor First Strand cDNA Synthesis Kit (04896866001, Roche, USA). The target genes were amplified and quantified in a LightCycler 480 SYBR Green 1 Master Mix (04707516001, Roche). The obtained data was analyzed with the 2−ΔΔCq method and the transcriptional level of target genes were uniformly normalized to GAPDH. The primers used in our study are presented in Supplementary Table1.

### 2.10 Determination of ROS

To assess the ROS level, the NRCMs which were seeded in 6-well plates were incubated with 2′,7′- dichlorodihydrofluorescein diacetate (DCFH-DA) for 0.5 h (37 °C). The ROS produced by the NRCMs was detected using an Olympus IX53 fluorescence microscope. The fluorescence intensity representing the level of ROS was measured using flow cytometry.

### 2.11 Immunofluorescence staining

The expression of specific proteins in NRCMs was performed by immunofluorescence staining as we described previously [21]. In detail, cell coverslips were fixed with 4% formaldehyde, and then permeabilized in 0.2% TritonX-100. Subsequently, cell coverslips were incubated with anti-STING (1:200), anti-IRF3 (1:100) or anti-NLRP3 (1:100) after adding 10% goat serum for 1 h at 37 °C. Then, the Alexa Fluor 488-goat anti-rabbit secondary antibody (1:200) was used to probe target proteins. DAPI was used to stain the cell nucleus of NRCMs or H9c2 cells. At last, we used Olympus DX51 fluorescence microscope (Tokyo, Japan) to observe their protein expression and nuclear translocation.

### 2.12 Co-Immunoprecipitation (IP) assay

The protein concentration of left ventricle tissues or NRCMs was determined by BCA protein concentration assay kit (Thermo Fisher Scientific, USA). 1 mg total protein lysates from each sample were used for immunoprecipitation (IP). The samples were incubated with rabbit polyclonal IgG control antibody, anti-IRF3 or anti-TXNIP. Then, the lysates were rotated for 4 h (4 °C). Subsequently, a total of 25 μl resuspended volume of Protein A/G PLUS-Agarose was added into the lysates and the mixture continued rotating for another 2 h. After washing and denaturing with immunoprecipitation buffer, the eluted proteins were immunoblotted with the anti-STING, anti-IRF3, anti-Trx, anti-TXNIP and anti-NLRP3 as descried in the above section.

### 2.13 Flow cytometry

To access apoptosis levels of different groups, the FITC-Annexin apoptosis detection kit (BD Biosciences, CA, USA) was used in our study following the standard instruction [18]. In brief, different groups of NRCMs were seeded in a 6-well plate and then challenged with LPS. After 6 h, NRCMs were harvested and washed with cold PBS 2 times. 1×binding buffer was used to resuspend cells. Whereafter, the cells were incubated in the dark with Annexin V/PI solution at room temperature for approximately 20 min. C6 Flow Cytometer™ system (BD Biosciences, CA, USA) was employed to analyze the apoptotic rate of NRCMs.

### 2.14 Statistical analyses

All data in our study were presented as the mean ± SEM. SPSS22.0 software was used to analyze the data. The differences between 2 groups were compared by Student's unpaired *t*-test. Multiple comparisons among different groups were analyzed by one-way ANOVA followed by a post hoc Tukey's test. Statistical significance was regarded as *P* < 0.05.

## 3 Results

### 3.1 The expression levels of STING and IRF3 in SIC

**Fig. 1:**
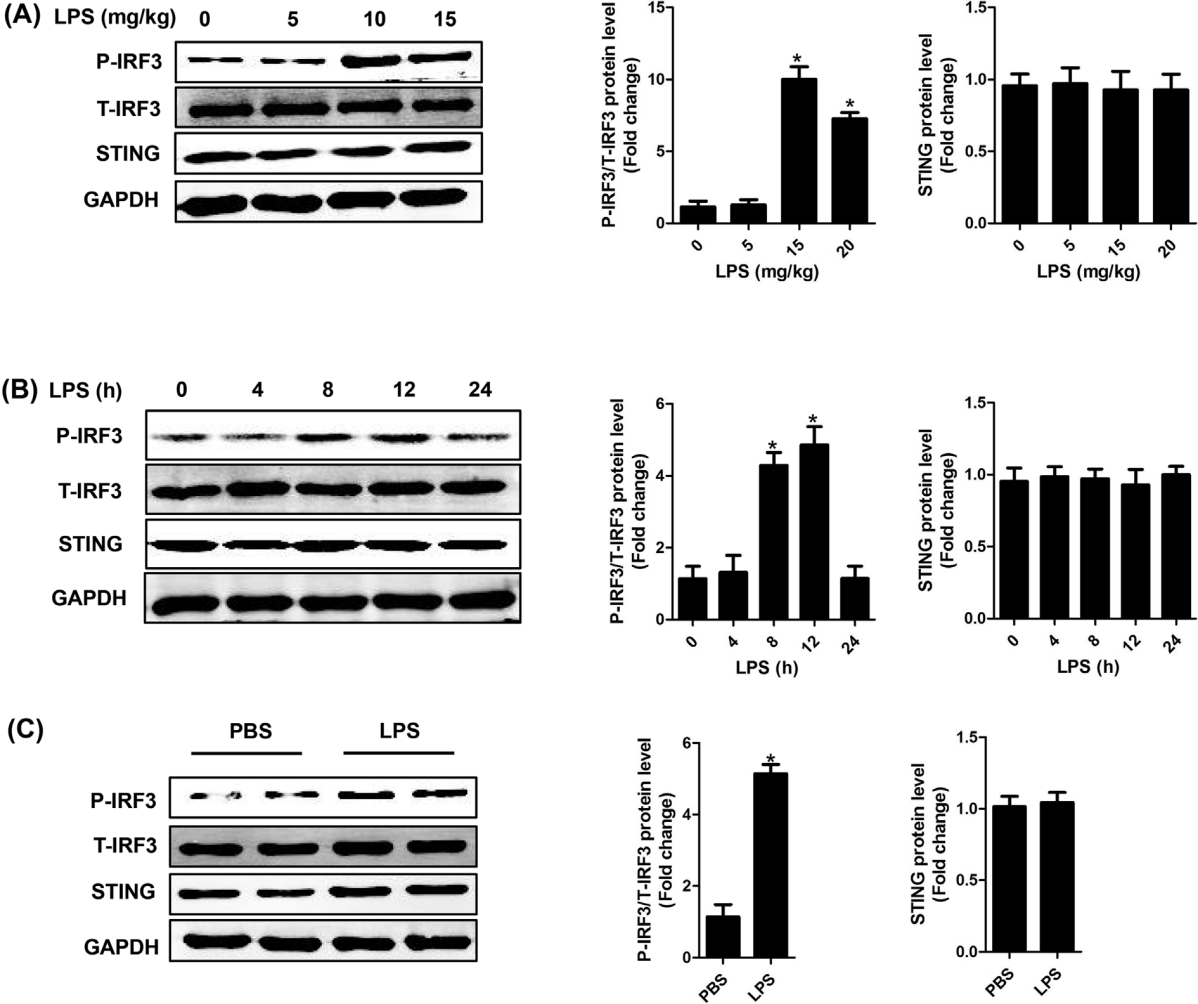
**The expression level of STING and IRF3 in SIC**. **A .** The protein levels of STING, P-IRF3 and T-IRF3 in mouse heart stimulated by different doses (ranging from 0 to 15 mg/kg) of LPS for 12 h in mice (n = 6, *P＜0.05 *vs.* 0 mg/kg group). **B.** The protein levels of STING, P-IRF3 and T-IRF3 in mouse heart stimulated by LPS (10 mg/kg) for 0, 4, 8, 12, and 24 h in mice (n = 6, *P＜0.05 *vs.* 0 h group). **C.** The protein levels of STING, P-IRF3 and T-IRF3 in NRCMs after LPS stimulation (1 μg/ml for 6 h) (n = 6, *P＜0.05 *vs.* PBS group).

Firstly, we detected the protein expression of STING, phosphorylated (P-) IRF3 and total (T-) IRF3 in normal heart tissues and heart tissues from mice challenged with LPS. The results showed that the protein expression level of STING was hardly affected by different doses of LPS (ranging from 0 to 15 mg/kg) for 12 h in mice (Fig. 1A). The protein level of P-IRF3 was very low when the dose of LPS was less than 5 mg/kg, but it was significantly increased when the dose of LPS was over 10 mg/kg. In particular, the level of P-IRF3 reached a peak value at the dose of 10 mg/kg. Subsequently, we observed the protein level of P-IRF3 at different time points after LPS injection (10 mg/kg) in mice. We found that the protein expression level of STING was still not altered at different time points. By contrast, the level of IRF3 at 8 h and 12 h was significantly increased, but it dropped to the baseline level at 24 h (Fig. 1B). Hence, the intervention time and dose of LPS in mice model was set as 10 mg/kg for 12 h. Furthermore, we confirmed the expression levels of STING and IRF3 in NRCMs challenged with LPS (1 μg/ml) for 6 h in vitro (Fig. 1C). These results indicated that STING-IRF3 may participate in the development of SIC.

### 3.2 LPS affected the intracellular location of STING and IRF3

**Fig. 2:**
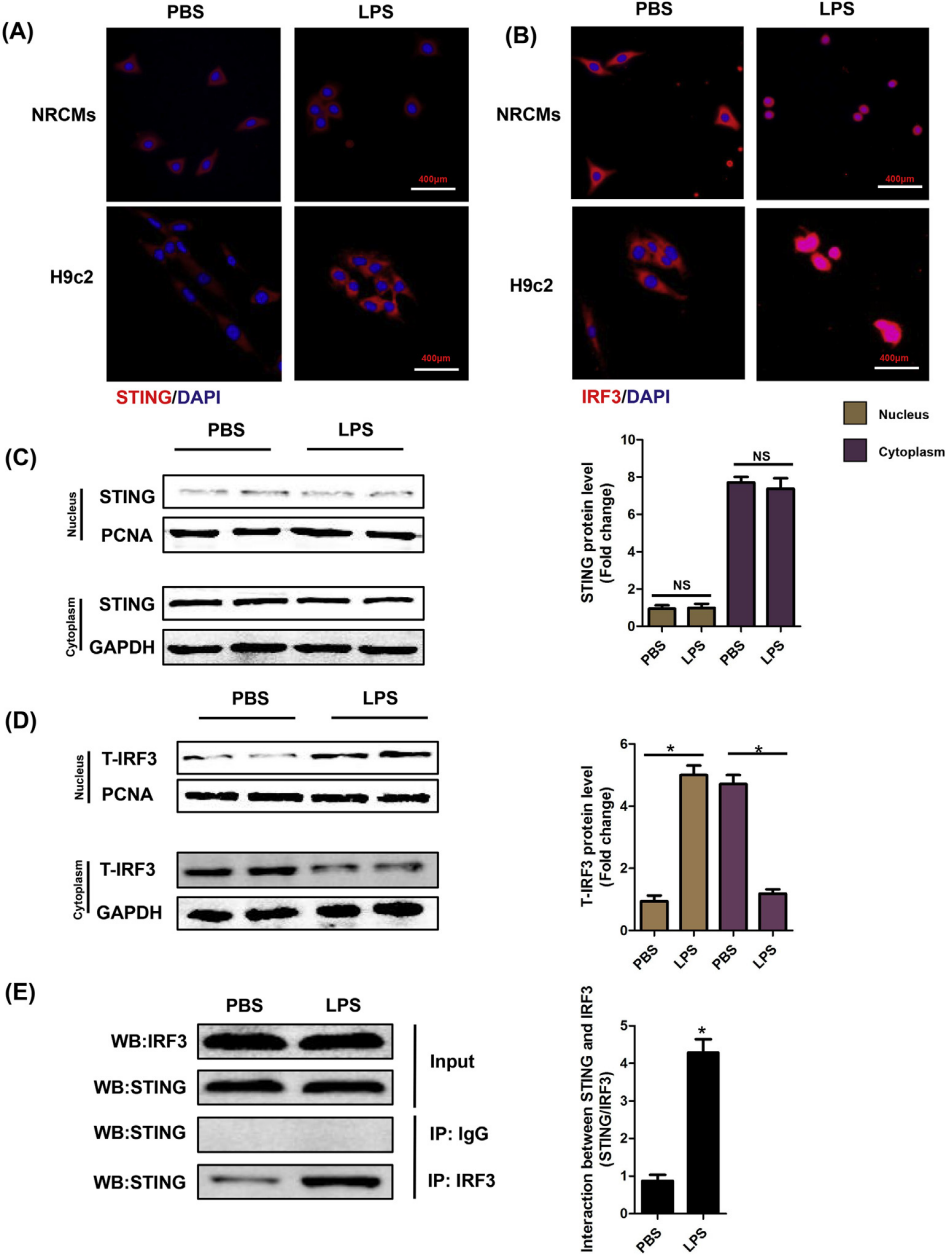
**LPS affected the intracellular location of STING and IRF3**. **A-B.** NRCMs or H9c2 cells were stimulated by LPS for 6 h. **A.** Representative images of immunofluorescence of STING in NRCMs and H9c2 cells (n = 6). **B.** Representative images of immunofluorescence of IRF3 in NRCMs and H9c2 cells (n = 6). **C.** The cytoplasm protein and nucleus protein levels of STING in NRCMs stimulated by LPS for 6 h (n = 6, *P＜0.05 *vs.* indicated group, NS, no significance). **D.** The cytoplasm protein and nucleus protein levels of T-IRF3 in NRCMs stimulated by LPS for 6 h (n = 6, *P＜0.05 *vs.* indicated group). **E.** Representative images of IP of STING and IRF3 in NRCMs stimulated by LPS for 6 h (n = 6, *P＜0.05 *vs.* PBS group).

We also observed the intracellular location of STING and IRF3 in NRCMs and H9c2 cells. Immunofluorescence analyses disclosed that LPS obviously triggered the perinuclear translocation of STING (Fig. 2A) and promoted the nuclear translocation of IRF3 (Fig. 2B). Furthermore, we detected the protein expression of STING and IRF3 in nucleus and cytoplasm of NRCMs, respectively. The results showed that STING is hardly expressed in nucleus and is consistently expressed in cytoplasm, regardless of PBS or LPS stimulation (Fig. 2C), which was consistent with our immunofluorescence analyze. By contrast, LPS could significantly induce the nuclear translocation of IRF3 but not affect the total expression of IRF3 in NRCMs (Fig. 2D). Co-Immunoprecipitation assay unveiled that IRF3 could interact with STING, which was in line with previous study (Fig. 2E) [22]. Considering that LPS is classical activator of toll-like receptor 4 (TLR4) [23], we used TLR4 siRNA to knock down the expression of TLR4 in NRCMs. We found that TLR4 inhibition in NRCMs counteracted the perinuclear translocation of STING triggered by LPS although TLR4 inhibition had no effects on the expression level of STING (Supplementary Fig. 1A). Additionally, we used Pam3Cys-Ser-(Lys)4, (TLR1/TLR2 agonist) to activate the TLR1 and TLR2 of NRCMs to observe its effects on STING activation. The results showed that Pam3Cys-Ser-(Lys)4 did not influence the expression or activation of STING (Supplementary Fig. 1B). These results indicated that LPS-induced STING activation is mediated by TLR4, but not TLR1 or TLR2.

### 3.3 STING knockdown inhibited LPS-induced phosphorylation and nuclear translocation of IRF3

**Fig. 3:**
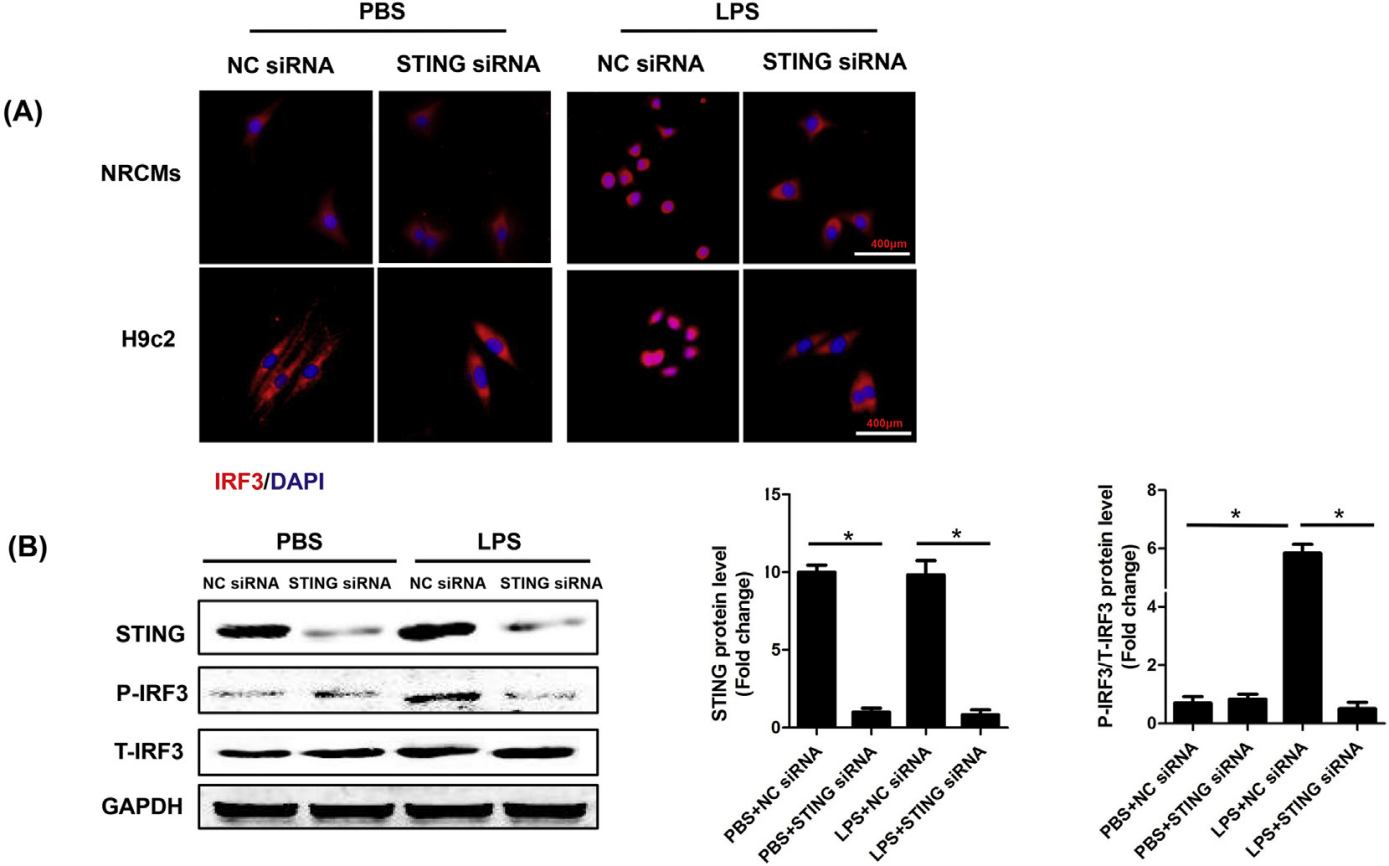
**STING knockdown inhibited LPS-induced phosphorylation and nuclear translocation of IRF3**. **A .** Representative images of immunofluorescence of IRF3 in NRCMs or H9c2 cells transfected with STING siRNA and then stimulated by LPS for 6 h. **B.** The protein levels of STING, P-IRF3 and T-IRF3 in NRCMs stimulated by LPS for 6 h (n = 6, *P＜0.05 *vs.* indicated group).

To assess the regulatory effect of STING on IRF3 in LPS-induced cardiac injury, the NRCMs and H9c2 cells were transfected with small interfering RNA (siRNA) to downregulate STING. As indicated in Fig. 3A, STING knockdown blocked LPS-induced perinuclear translocation of IRF3 in NRCMs and H9c2 cells. Immunoblot analysis (Fig. 3B) also uncovered that STING knockdown could inhibit LPS-induced phosphorylation of IRF3. These results further proved potential regulatory effect of STING on IRF3 in SIC.

### 3.4 STING deficiency improved survival and cardiac function of LPS-treated mice

**Fig. 4:**
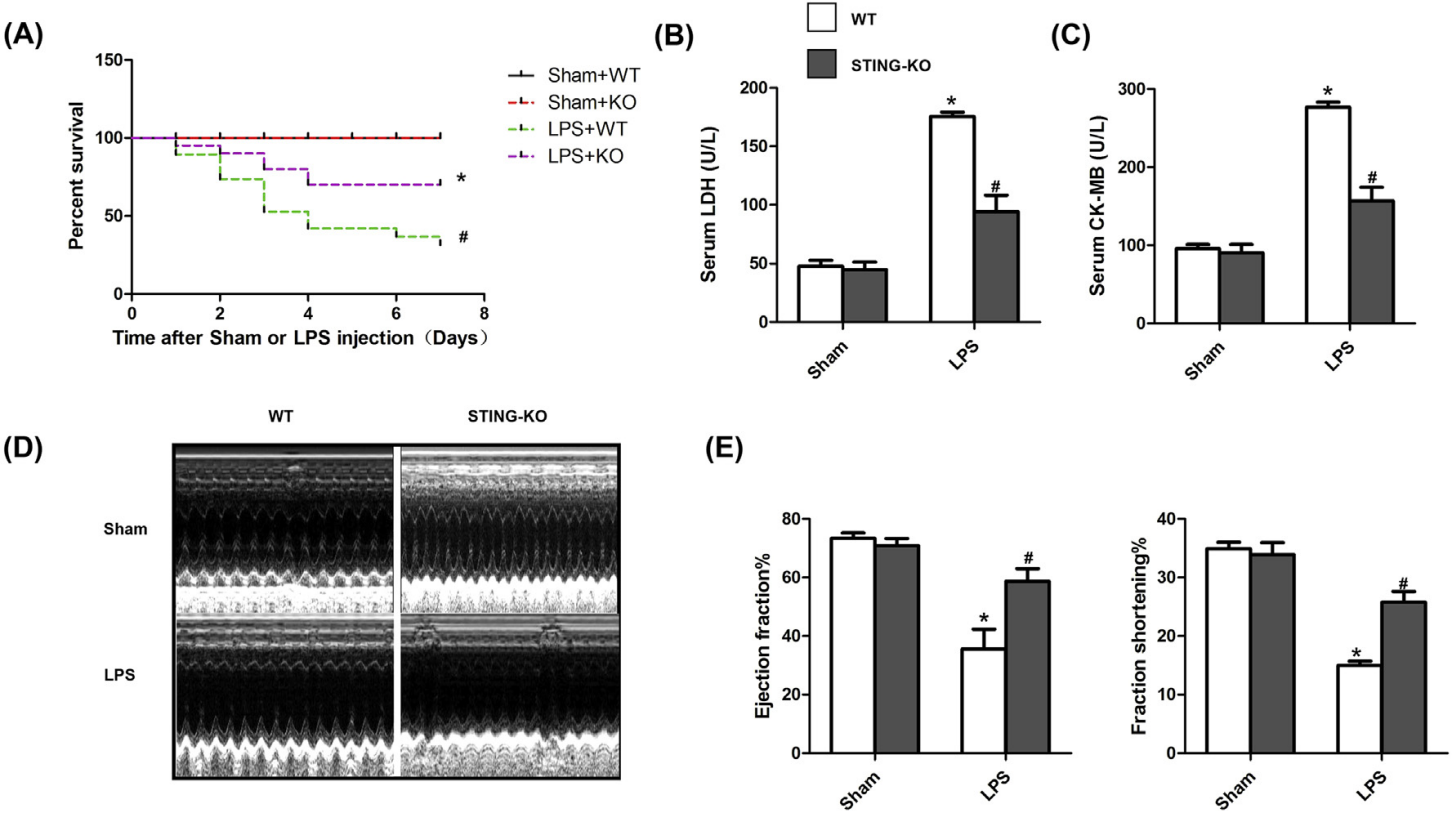
**STING deficiency improved survival and cardiac function of LPS-treated mice**. **A .** Effect of STING deficiency on the 7-day survival rate after LPS injection (n = 10). **B.** Effect of STING deficiency on LPS-induced LDH release (n = 6). **C.** Effect of STING deficiency on LPS-induced CK MB release (n = 6). **D.** Representative echocardiographic images of each group (n = 6). **E.** Effect of STING deficiency on left ventricle ejection fraction and left ventricle fractional shortening of each group after Sham or LPS injection (n = 6). *P < 0.05 *vs.* Sham + WT, #P < 0.05 *vs.* LPS + WT.

Subsequently, we observed the roles of STING in survival, cardiac injury and cardiac function of LPS-treated mice. As shown in Fig. 4A, we calculated the 7-day survival rate in the following 4 groups, including Sham + WT group, Sham + STING KO group, LPS + WT group and LPS + STING KO group. The 7-day percent survival in Sham + WT group and Sham + STING KO group were almost 100%. However, 7 days after LPS injection, there was a significant decline in the percent survival in LPS + WT group (40%) (versus Sham + WT group, P＜0.05). With the knockout of STING in mice, the percent survival in LPS + STING KO group went up to 75% (versus LPS + WT group, P＜0.05). LDH release is a sensitive biomarker of cardiac injury, so we detected the levels of LDH and CK MB in the serum. As shown in Fig. 4B&C, STING deficiency significantly reversed the increased level of LDH and CK MB in LPS-treated mice. Using echocardiography, we also investigated cardiac function in 4 groups. As shown in Fig. 4D&E, STING deficiency significantly improved cardiac function of LPS-treated mice, as evidenced by increased ejection fraction and increased fraction shortening.

### 3.5 STING deficiency suppressed cardiac inflammation, apoptosis and pyroptosis of LPS-treated mice

**Fig. 5:**
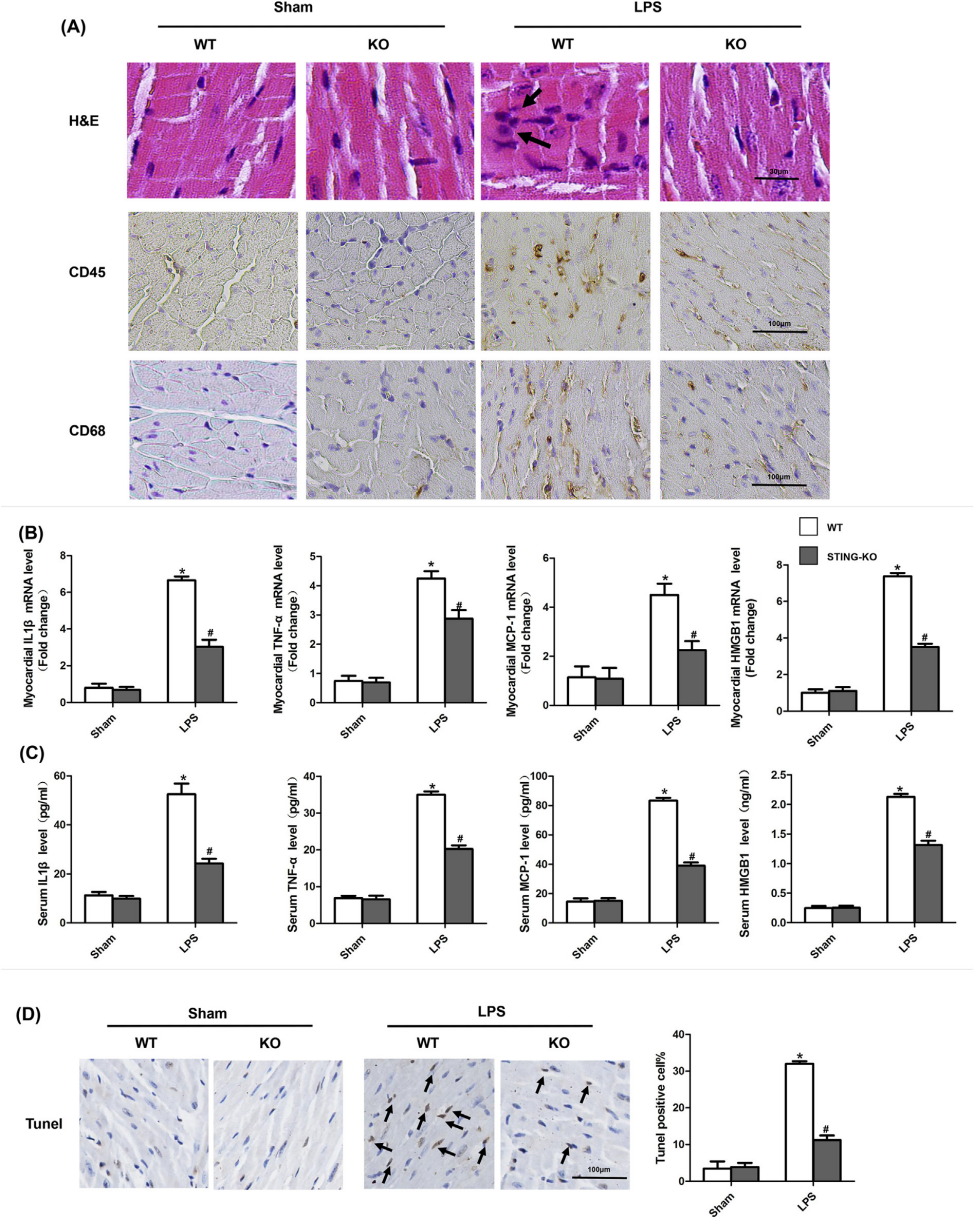

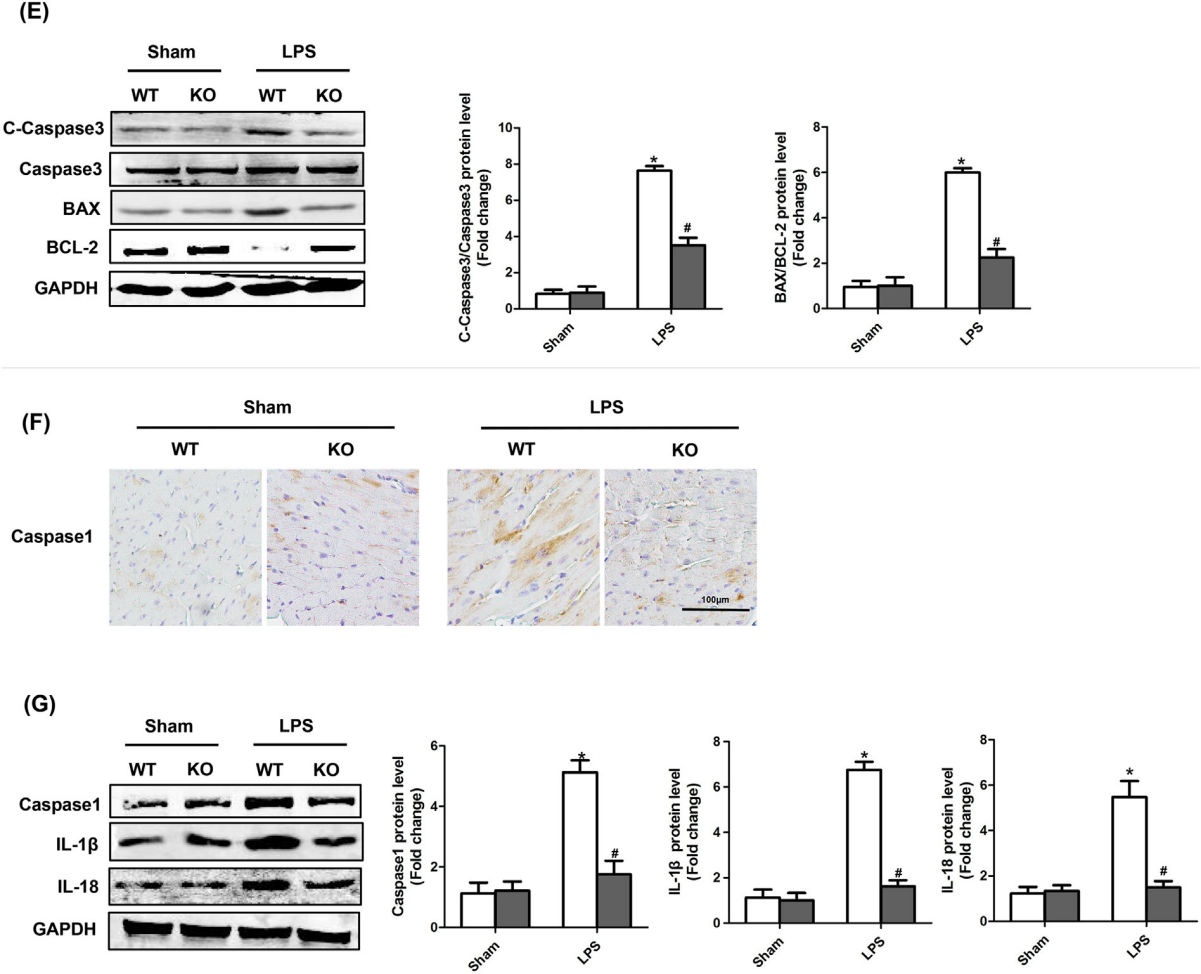
**STING deficiency suppressed cardiac inflammation, apoptosis and pyroptosis of LPS-treated mice**. **A .** Representative images of the morphological analysis and inflammatory cells infiltration as reflected by the H&E staining, and immunohistochemistry staining for CD45 and CD68 protein (n = 6, 10 + fields per heart). The arrow showed inflammatory cells. **B.** The levels of inflammatory cytokines including IL-1β, TNF-α, MCP-1 and HMGB1 in myocardial tissues of each group after Sham or LPS injection (n = 6). **C.** The levels of inflammatory cytokines including IL-1β, TNF-α, MCP-1 and HMGB1 in serum of each group after Sham or LPS injection (n = 6). **D.** TUNEL staining of each group after Sham or LPS injection (n = 6, 10 + fields per heart). The arrow indicated Tunel positive cells. **E**. The protein levels of C-Caspase3, Caspase3, BAX and BCL-2 of each group after Sham or LPS injection (n = 6). **F.** Representative images of the immunohistochemistry staining for Caspase1 (n = 6, 10 + fields per heart). **E**. The protein levels of Caspase1, IL-1β and IL-18 of each group after Sham or LPS injection (n = 6). *P < 0.05 *vs.* Sham + WT, #P < 0.05 *vs.* LPS + WT.

The myocardial sections were stained with H&E and immunohistochemistry to assess myocardial tissue damage and inflammatory cells infiltration (Fig. 5A). The photomicrographic sections of LPS + WT group showed obvious inflammatory cells infiltration. But in LPS + STING KO group, the cardiac muscle fibers had clear striation and few inflammatory cells infiltrated in myocardial tissue. Immunohistochemical staining showed that STING deficiency reduced the infiltration of CD45-labeled leukocytes and CD68-labeled macrophages in myocardial tissue of LPS-treated mice. Meanwhile, we measured the levels of pro-inflammatory cytokines in both myocardial tissue and serum in 4 groups. As displayed in Fig. 5B&C, the serum and myocardial levels of IL-1β，TNF-α，MCP-1 and HMGB1 were significantly increased in LPS + WT group compared with Sham + WT group. However, STING deficiency reduced the levels of above pro-inflammatory cytokines. Also, we evaluated the apoptosis levels in 4 groups. As expected (Fig. 5D&E), STING deficiency significantly reduced the level of apoptotic cardiomyocytes in LPS-treated mice, as evidenced by Tunel staining, decreased ratio of C-Caspase3 to T-Caspase3 and decreased ratio of BAX to BCL-2. Finally, we detected the markers of pyroptosis in 4 groups. As indicated in Fig. 5F&G, STING knockout also significantly inhibited the protein levels of Caspase1, IL-1β and IL-18, which could initiate pyroptosis. Taken together, these results demonstrated that STING deficiency may exert cardioprotection by suppressing cardiac inflammation, apoptosis and pyroptosis in LPS-treated mice.

### 3.6 STING triggered the activation of NLRP3 inflammasome in heart of LPS-treated mice

**Fig. 6:**
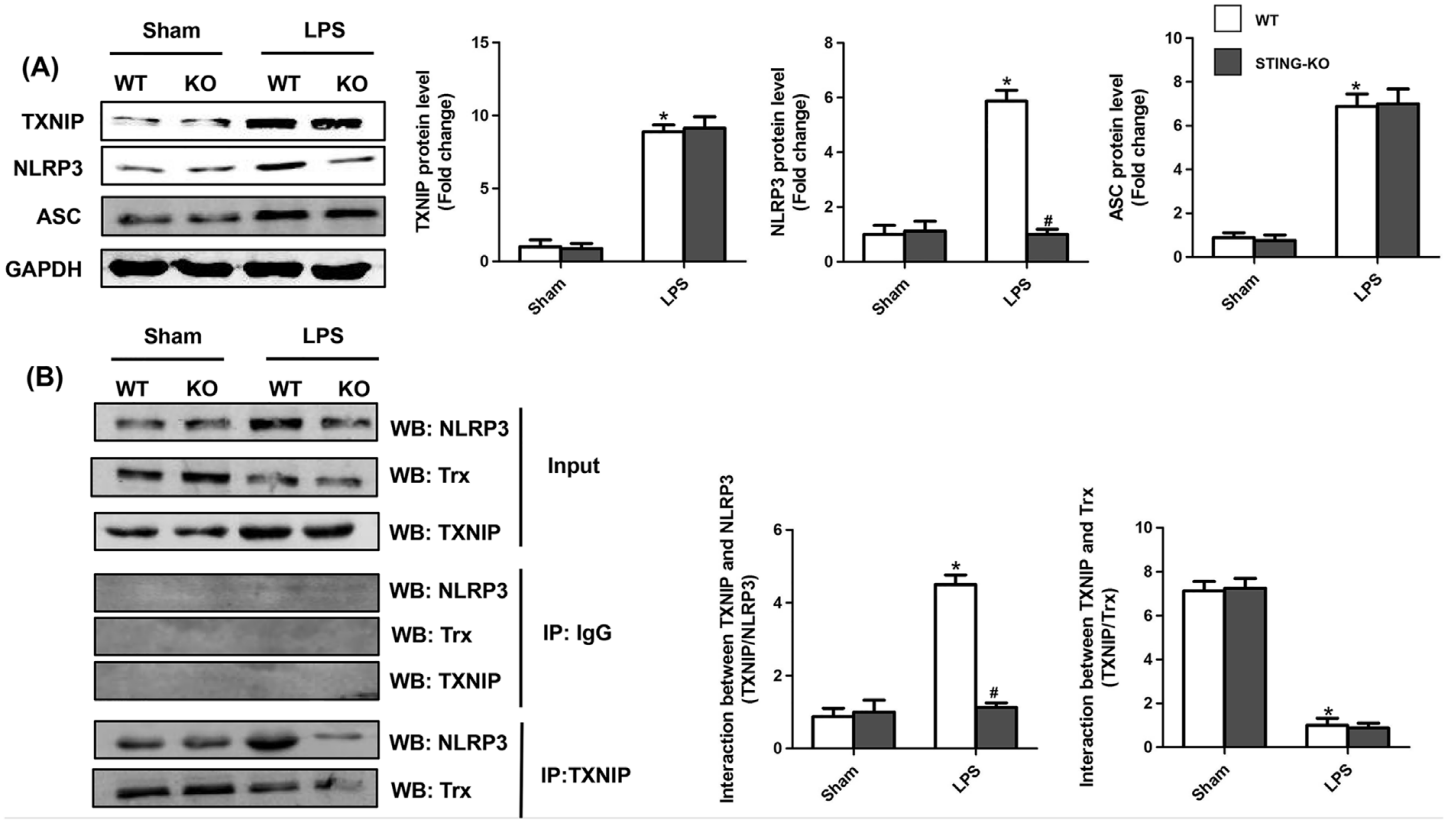
**STING triggered the activation of NLRP3 inflammasome in heart of LPS-treated mice**. **A .** The protein levels of TXNIP, NLRP3 and ASC of each group after Sham or LPS injection (n = 6). **B.** Representative images of IP of NLPR3, TXNIP and Trx in each group after Sham or LPS injection (n = 6). *P < 0.05 *vs.* Sham + WT, #P < 0.05 *vs.* LPS + WT.

In this study, myocardial levels of TXNIP, NLRP3 and ASC were significantly increased in mice after LPS injection. Intriguingly, STING deficiency only decreased the level of NLRP3, but had no effects on the expression of TXNIP and ASC in SIC (Fig. 6A). To further explore the effects of STING deficiency on myocardial NLRP3 inflammasome in LPS-treated mice, we performed CO-IP assays and discovered that inhibition of STING decreased interaction of TXNIP and NLRP3, While STING deficiency did not enhance the interaction of TXNIP and Trx (Fig. 6B). A direct reason resulting in the decreased interaction of TXNIP and NLRP3 was that STING deficiency reduced the protein level of NLRP3, therefore inhibiting the formation of TXNIP/NLRP3 complex.

### 3.7 STING activated NLRP3 in an IRF3-dependent manner in LPS-induced NRCMs

**Fig. 7:**
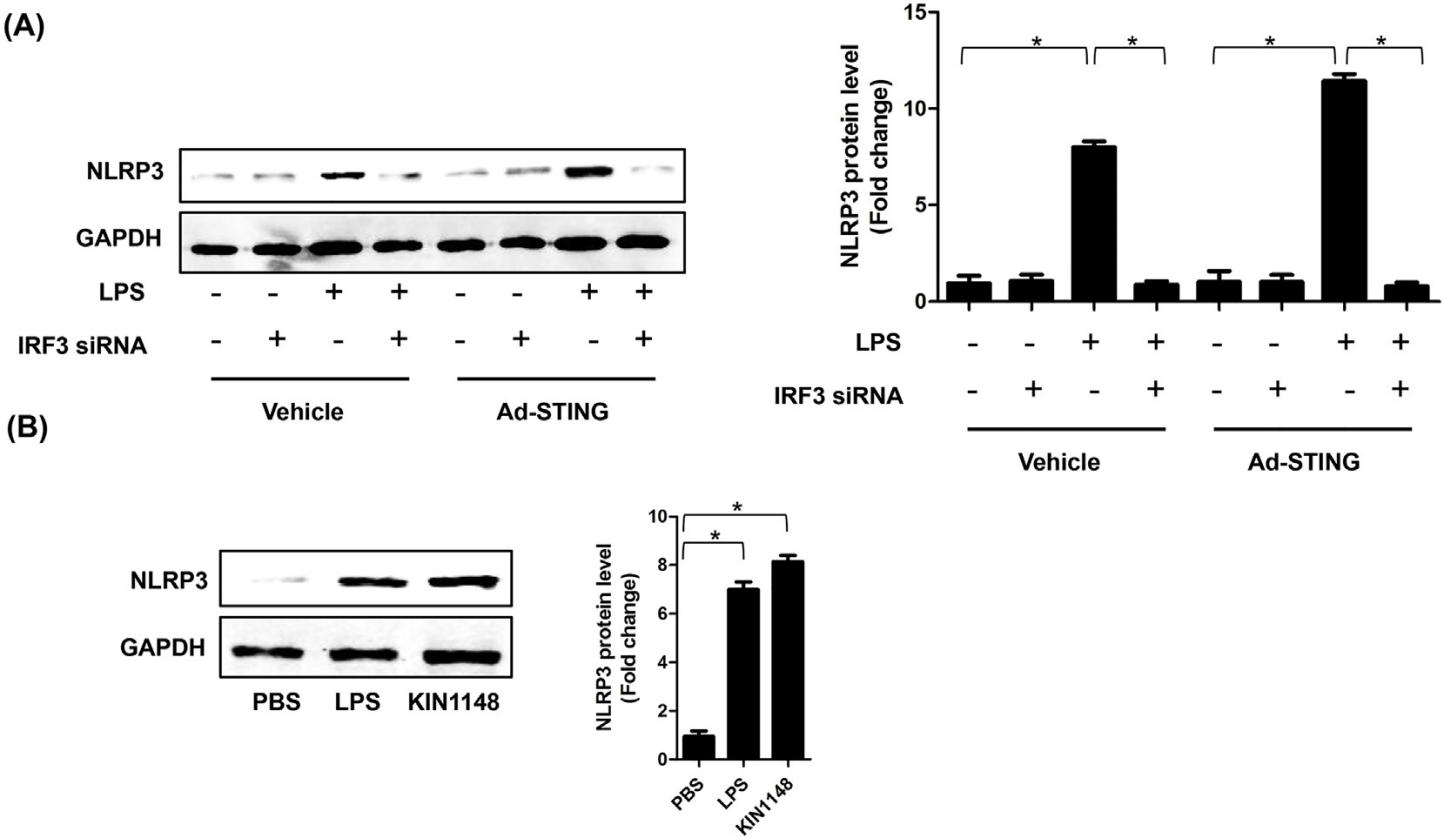
**STING activated NLRP3 in an IRF3-dependent manner in LPS-induced NRCMs**. **A .** The protein levels of NLRP3 in indicated groups that were treated with IRF3 siRNA and (or) Ad-STING and then stimulated with LPS for 6 h (n = 6). **B .** IRF3 agonist, KIN1148 increased the protein expression level of NLRP3 (n = 6). *P＜0.05 *vs.* indicated group.

Whereafter, we explored whether the upregulation of NLRP3 by STING dependens on the IRF3 activation. We used adenovirus (Ad-) to overexpress NLRP3 and siRNA to knock down IRF3 in NRCMs stimulated by LPS. As shown in Fig. 7A, IRF3 silencing significantly inhibited LPS-induced NLRP3 expression in NRCMs even though STING was overexpressed. Additionally, we employed KIN1148a, small-molecule IRF3 agonist, to activate IRF3 in NRCMs. As expected (Fig. 7B), KIN1148 treatment significantly upregulated the expression of NLRP3 in NRCMs, which further demonstrated that IRF3 activation by STING could increased the expression of NLRP3 in NRCMs.

### 3.8 STING induced inflammation, apoptosis and pyroptosis in LPS-induced cardiomyocytes by activating NLRP3

**Fig. 8:**
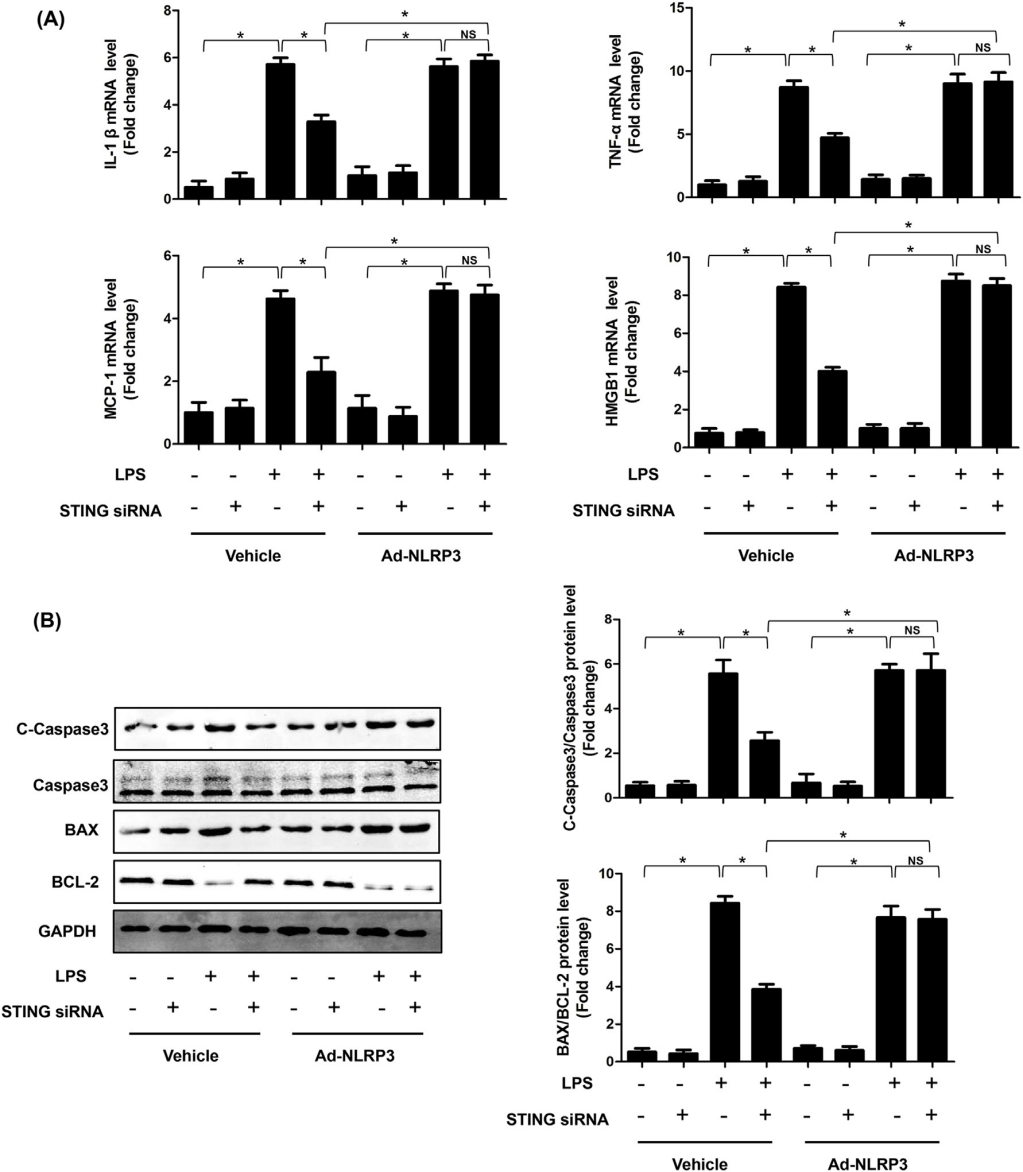

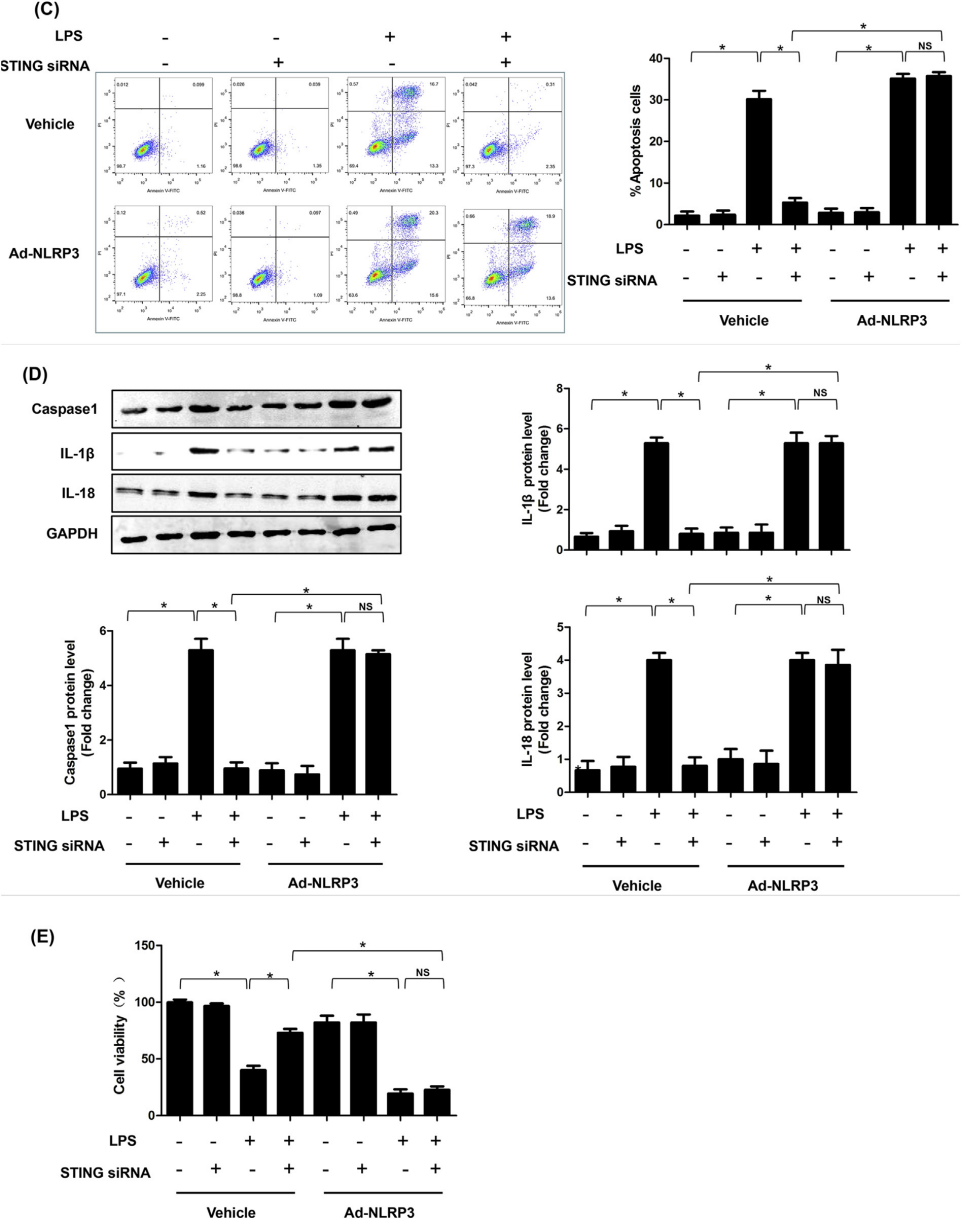
**STING induced inflammation, apoptosis and pyroptosis in LPS-induced cardiomyocytes by activating NLRP3**. **A .** The mRNA levels of IL-1β, TNF-α, MCP-1 and HMGB1 in indicated groups that were treated with STING siRNA and (or) Ad-IRF3and then stimulated with LPS for 6 h (n = 6). **B.** The protein levels of C-Caspase3, Caspase3, BAX and BCL-2 in indicated groups (n = 6). **C.** Apoptosis analysis by flow cytometry in indicated groups (n = 6). **D**. The protein levels of Caspase1, IL-1β and IL-18 in indicated groups (n = 6). **E.** Cell viability was detected by an CCK8 assay in indicated group (n = 6). *P＜0.05 *vs.* indicated group.

We also investigated the role of STING-mediated NLRP3 activation in LPS-induced cardiac injury in vitro. As expected, STING silencing significantly suppressed the mRNA expression levels of IL-1β, TNF-α, MCP-1 and HMGBA in NRCMs stimulated by LPS for 6 h, however, the levels of which were increased after NLRP3 overexpressed (Fig. 8A). Additionally, STING silencing displayed obvious anti-apoptosis effects in LPS-treated NRCMs. Similarly, the upregulation of NLRP3 offset the anti-apoptosis effect of STING silencing (Fig. 8B–C). Finally, we observed the effect of STING-mediated NLRP3 activation on pyroptosis of NRCMs. We detected the protein expression levels of Caspase1, IL-1β and IL-18. As shown in Fig. 8D, knockdown of STING significantly blocked pyroptosis of LPS-treated NRCMs. But STING silencing-induced pyroptosis inhibition was initiated by NLRP3 overexpression. These data directly indicate that STING-IRF3-mediated NLRP3 activation triggered inflammation, apoptosis and pyroptosis in LPS-treated NRCMs.

### 3.9 ROS is essential for cytoplasmic translocation of NLRP3 in LPS-induced NRCMs

**Fig. 9:**
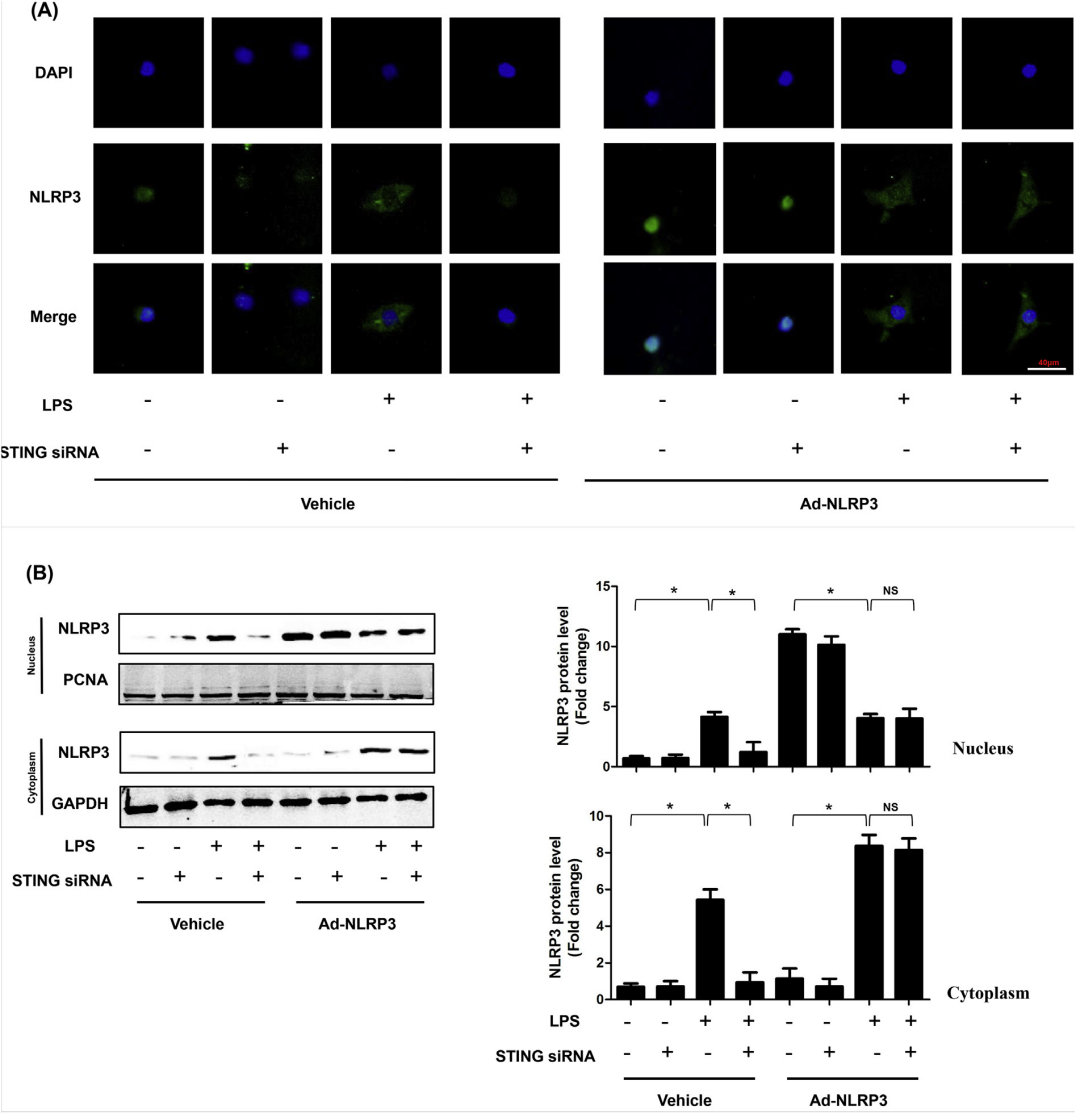

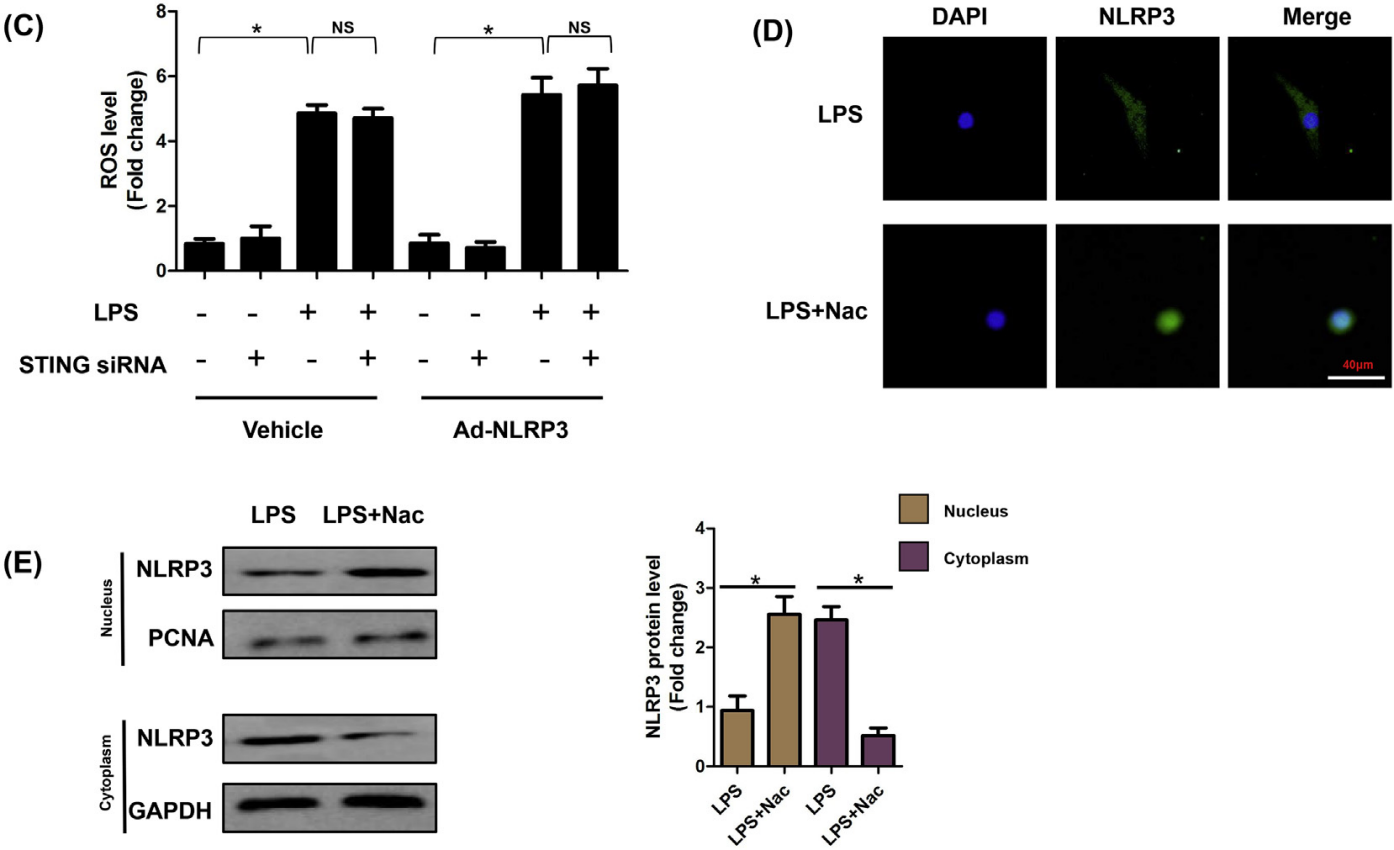
**ROS is essential for cytoplasmic translocation of NLRP3 in LPS-induced NRCMs**. **A .** Representative images of immunofluorescence of NLRP3 in NRCMs in indicated groups (n = 6). **B.** The cytoplasm protein and nucleus protein levels of NLPR3 in NRCMs in indicated groups (n = 6). **C.** The ROS levels of NRCMs in indicated groups (n = 6). **D.** ROS scavenger, Nac blocked the cytoplasmic translocation of NLRP3 (n = 6). E. The cytoplasm protein and nucleus protein levels of NLPR3 in NRCMs in NRCMs treated with Nac (n = 6). *P＜0.05 *vs.* indicated group, NS, no significance.

ROS played vital roles in inflammasome activation. On the one hand, ROS could promote the dissociation of TXNIP-Trx protein complex. On the other one hand, ROS triggered the association of NLRP3 with TXNIP [8]. Here, we firstly detected the regulatory role of STING in cytoplasmic translocation of NLRP3. The results (Fig. 9A&B) showed that STING could upregulate the expression of NLRP3 under the stimulation of LPS. But STING was not responsible for cytoplasmic translocation of NLRP3. Intriguingly, we found that the nuclear export of NLRP3 to cytoplasm could be triggered by LPS, whereupon we detected the ROS levels in the indicated groups. As expected, LPS could trigger the generation of ROS regardless of STING or NLRP3 expression (Fig. 9C). Hence, we used N-acetyl-l-cysteine (Nac), which was an inhibitor of cellular ROS, to observe the regulatory effects of ROS on cytoplasmic translocation of NLRP3. As shown in (Fig. 9D–E), The elimination of ROS blocked LPS-induced cytoplasmic translocation of NLRP3.

## 4 Discussion

**Fig. 10:**
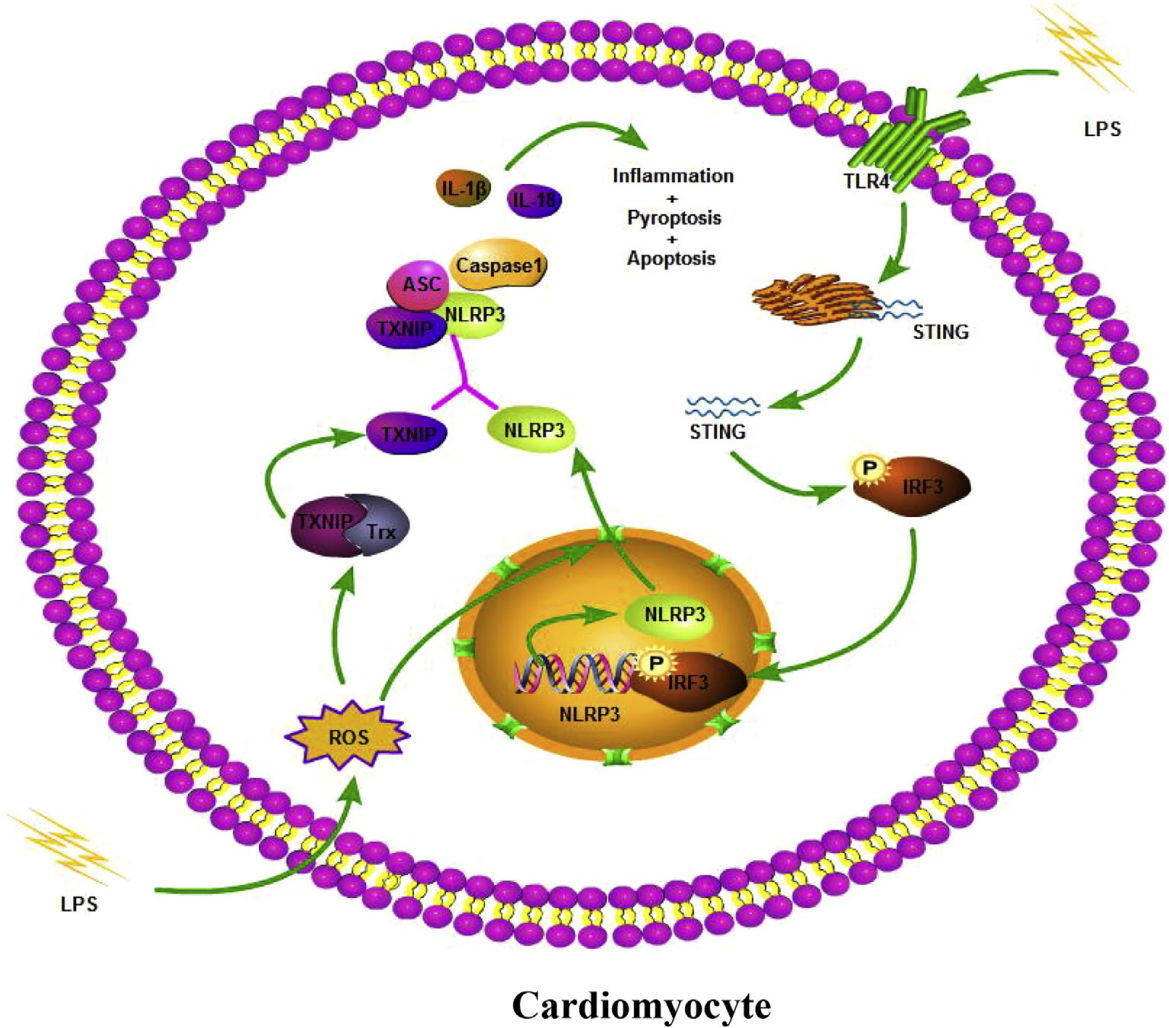
A putative scheme illustrating the mechanism by which STING-IRF3 contributes to inflammation, pyroptosis and apoptosis of cardiomyocytes in LPS-induced cardiac injury.

Here, to the best of our knowledge, this was the first study to disclose that STING exerted a previously unreported role in LPS-induced SIC. Our findings demonstrated that STING-IRF3 could trigger LPS-induced cardiac dysfunction, inflammation, apoptosis and pyroptosis by activating NLRP3 in mice. STING knockout could suppress the phosphorylation and nuclear translocation of IRF3, which further inhibited NLRP3-mediated inflammation, apoptosis and pyroptosis of cardiomyocytes. We also provided evidence that LPS-induced ROS generation in cardiomyocytes played an essential role in the export of NLRP3 from the nucleus to the cytoplasm, which was independent of the expression of STING (Fig. 10).

STING (also known as MPYS, MITA, TMEM173 and ERIS) is a multispanning transmembrane protein, which was ubiquitously existed in endoplasmic reticulum, plasma membrane, as well as the surface of the outer mitochondrial membrane. Originally, STING was characterised as a growth inhibitor mediating B lymphoma cells death induced by anti-MHC class II antibody [[24], [25], [26]]. IRF3, which ubiquitously resides in the cytoplasm, is in essence a constitutively expressed transcription factor [27]. Once STING is activated by viral infection, mitochondrial DNA or endoplasmic reticulum stress, it could recruit TANK-binding kinase 1 (TAK1) and IRF3, and then phosphorylate IRF3 by TAK1. P-IRF3 subsequently dimerizes and translocates to the nucleus, regulating the expression of various inflammatory factors [[28], [29], [30]]. More recently, STING-IRF3 was proved to play vital roles in endothelial inflammation caused by free fatty acid, and STING knockout could partially protect against adipose tissue inflammation, insulin resistance, obesity, and glucose intolerance in obese mice [22]. In myocardial infarction, interruption of STING-IRF3 signaling not only gave rise to reduced levels of chemokines, inflammatory cytokines, or inflammatory cell infiltration in cardiac tissues, but also improved cardiac function [31]. In our study, we demonstrated that in the development of SIC induced by LPS, the protein expression of STING maintained all along while the level of P-IRF3 increased at 8–12 h after LPS (10 mg/kg) injection in vivo. Furthermore, LPS stimulation could promote the perinuclear translocation of STING and the nuclear translocation of IRF3, indicating that STING-IRF3 participated in the pathology of SIC. And STING deficiency improved cardiac function and survival condition of mice undergoing LPS insult. Additionally, STING inhibition also ameliorated inflammation, cardiomyocyte pyroptosis and apoptosis in mice after LPS injection or LPS-treated NRCMs.

NLRP3 is a vital mediator in the initiation of the immune response and formation of inflammasome, which could be induced by various host ‘danger’, such as DAMPs and PAMPs. Once activated, NLRP3 inflammasome promotes the maturation and secretion of pro-inflammatory cytokines, such as Caspase1, IL-1β as well as IL-18 and initiates pyroptosis. In sepsis, pyroptosis triggered by inflammasome could rupture the cell membrane, resulting in releases of multiple inflammatory factors [32]. In mice with SIC, NLRP3 detection in cardiomyocytes reduced the release of IL-1β in a NLRP3-dependent manner and the release of IL-6 in a NLRP3-independent manner [10]. In our study, we found that the expression levels of NLRP3 in both cardiac tissue and cardiomyocytes were increased after LPS stimulation. Furthermore, we found that the expression of NLRP3 could be activated in a STING-IRF3 dependent manner in SIC. The protective effects of STING deficiency could be offset by NLPR3 overexpression. NLRP3 expression facilitated LPS-induced pyroptosis by secreting Caspase1, IL-1β as well as IL-18 in cardiomyocytes. Previous study also unveiled that the cGAS-STING-induced NLRP3 inflammasome was implicated with viral and bacterial sensing, which constituted the vital DNA-Sensing inflammasome in human myeloid cells [13]. To our knowledge, NLRP3 inflammasome activation directly promoted the production of IL-1β, IL-18 and Caspase1 [33]. However, in our study, we observed that LPS also promoted the production of other inflammatory factors including TNF-α, MCP-1 and HMGB1 in a NLRP3 dependent manner. Dora Lippai，s study found that increased secretion of IL-1β byNLRP3 inflammasome activation could further amplify expression of inflammatory mediators (TNF-α, MCP-1 and HMGB1) in the brain of mice after chronic alcohol administration [34]. In a pulmonary infection model, the expression of HMGB1 in macrophage was also associated with NLRP3 and ASC [11]. Here, we speculate that the production of TNF-α, MCP-1 and HMGB1 in serum and myocardial tissues may be caused by the IL-1β, which further amplified the inflammation in heart in a NLRP3 dependent manner. Another interesting result in our study was that LPS also induced cardiomyocytes apoptosis in a NLRP3 inflammasome activation manner. As we know, Caspase1 generated by NLRP3 inflammasome is involved in rapid lytic cell death termed pyroptosis, which may be not responsible for apoptosis. V Sagulenko et al. reported that NLRP3 inflammasome could also activate Caspase8, apart from Caspase1-dependent pyroptosis. Activated Caspase8 promoted Caspase3 cleavage in response to nigericin and initiated apoptosis [13]. Consistent with their study, we also found that the expression of C-Caspase3 was positively regulated by NLRP3 inflammasome activation in LPS-treated cardiomyocytes, indicating that Caspase8/3 induced apoptosis can be also regarded as an intrinsic part of inflammasome function.

The NLRP3 inflammasome could be activated by various danger stimuluses which derive not only from metabolic dysregulation (DAMPs) but also from microorganisms (PAMPs) [[35], [36], [37]]. At present, a total of 3 models for activation of the NLRP3 inflammasome have been put forward, namely the ROS model [38,39], the K^+^ efflux model [40], and the lysosome rupture model [41,42]. Compared with the latter two models, the ROS model was more general for the activation of NLRP3. Various NLRP3 activator involving ATP and particulate activators (asbestos inducing K^+^ efflux and silica that triggering lysosome rupture), could cause the production of short-lived ROS, while treatment with ROS scavengers significantly suppressed the activation of NLRP3 in response to the NLRP3 agonists listed above [8]. Additionally, treatment with NLRP3 agonists also triggered the dissociation of TXNIP/Trx complex and the association of NLRP3 with TXNIP in a ROS-dependent manner, leading to the activation of NLRP3 inflammasome [43]. In human myeloid cells stimulated by ultrapure *E. coli* LPS, STING translocated to lysosomes once it was activated, which contributed to lysosome rupture and cell death. The subsequent drop in cytoplasmic K^+^ then resulted in the classical activation mode of NLRP3, eventually inducing pyroptosis [13]. In our study, we found that LPS treatment significantly increased the generation of ROS in cardiomyocytes, which was independent of STING-IRF3 activation. The excessive ROS promoted the export of NLRP3 from the nucleus to the cytoplasm.

In conclusion, this was the first study to disclose that STING knockout could attenuate LPS-induced cardiac injury by inhibiting NLRP3-mediated inflammation, apoptosis and pyroptosis. Our study provides basic evidence that targeting STING-IRF3 may serve as a therapeutic strategy for sepsis-induced cardiomyopathy.

## Conflicts of interest

The authors declare no conflicts of interest.

## Sources of funding

This work was supported by grants from the National Natural Science Foundation of China (No. 81770399, 81470516, 81530012, 81700353), the Fundamental Research Funds for the Central Universities of China (2042018kf0121), National Key R&D Program of China (2018YFC1311300) and Development Center for Medical Science and Technology, National Health and Family Planning Commission of the People's Republic of China (The prevention and control project of cardiovascular disease, 2016ZX-008-01).

## Data availability

All data that support the findings in this study are available from the corresponding author upon reasonable request.

## Author contribution

Ning Li and Heng Zhou conceived the study design. Qizhu Tang and Wei Deng designed the experiments. Haiming Wu and Qingqing Wu analyzed the data. Ning Li and Mingxia Duan drafted the manuscript. Qingqing Wu reviewed and revised the manuscript.

## Figures and Tables

**figs1:**