# Supplementary material for: STING-IRF3 contributes to lipopolysaccharide-induced cardiac dysfunction, inflammation, apoptosis and pyroptosis by activating NLRP3
Source: Redox Biol. 2019 May 13;24:101215. doi: 10.1016/j.redox.2019.101215 (PMC6529775; doi:10.1016/j.redox.2019.101215)
Supplement: Multimedia component 1 [file mmc1.docx]

**Supplementary material**

**Table 1. Primers in real-time real time PCR.**

| **Gene** | **Species** | **Forward primer** | **Reverse primer** |
| --- | --- | --- | --- |
| IL-1β | Mouse | AATGAAGGAACGGAGGAGCC | CTCCAGCCAAGCTTCCTTGT |
| TNF-α | Mouse | ACTGAACTTCGGGGTGATCGGT | TGGTTTGCTACGACGTGGGCTA |
| MCP-1 | Mouse | TGGCTCAGCCAGATGCAGT | CCAGCCTACTCATTGGGATCA |
| HMGB1 | Mouse | CCGGCAAGTTTGCACAAAGA | TTGGGAGGGCGGAGAATCAA |
| GAPDH | Mouse | ACTCCACTCACGGCAAATTC | TCTCCATGGTGGTGAAGACA |
| IL-1β | Rat | CCTCACCCTGTTTGGGGTTT | GTTAGCATGCCTGCCCTGAA |
| TNF-α | Rat | AGCATGATCCGAGATGTGGAA | TAGACAGAAGAGCGTGGTGGC |
| MCP-1 | Rat | AGTCGGCTGGAGAACTACAAGA | CTGAAGTCCTTAGGGTTGATGC |
| HMGB1 | Rat | TGGGGGAAATTTGTAGTGAGGA | CAGGTCCACAGCCAGACTTT |
| GAPDH | Rat | GACATGCCGCCTGGAGAAAC | AGCCCAGGATGCCCTTTAGT |

**Supplementary figure1**

A. NRCMs were stimulated by LPS for 6h with or without TLR4 knockdown. Representative images of immunofluorescence of STING in NRCMs (n=6). The protein level of STING in NRCMs in indicated groups (n=6, *P＜0.05 vs. indicated group). B. NRCMs were stimulated by LPS for 6h with or without Pam3Cys-Ser-(Lys)4 (33ng/mL). Representative images of immunofluorescence of STING in NRCMs (n=6). The protein level of STING in NRCMs in indicated groups (n=6, *P＜0.05 vs. indicated group).
